# Phytochemistry, Anti-cancer, and Anti-diabetic Properties of Plant-Based Foods from Mexican Agrobiodiversity: A Review

**DOI:** 10.3390/foods13244176

**Published:** 2024-12-23

**Authors:** Adriana García-Gurrola, Ana Laura Martínez, Abraham Wall-Medrano, Francisco J. Olivas-Aguirre, Estefania Ochoa-Ruiz, Alberto A. Escobar-Puentes

**Affiliations:** 1 Faculty of Medicine and Psychology, Autonomous University of Baja California, Tijuana 22427, Baja California, Mexico; adriana.garcia.gurrola@uabc.edu.mx (A.G.-G.); ana.laura.martinez.martinez@uabc.edu.mx (A.L.M.); estefania.ochoa@uabc.edu.mx (E.O.-R.); 2 Biomedical Sciences Institute, Autonomous University of Ciudad Juárez, Ciudad Juaez 32300, Chihuahua, Mexico; awall@uacj.mx (A.W.-M.); javier.olivas@uacj.mx (F.J.O.-A.)

**Keywords:** anti-diabetic, anti-cancer, Mexican agrobiodiversity, plant-based foods, phytochemicals

## Abstract

Type 2 diabetes mellitus (T2DM) and cancer are significant contributors to morbidity and mortality worldwide. Recent studies have increasingly highlighted the potential of phytochemicals found in plants and plant-based foods for preventing and treating these chronic diseases. Mexico’s agrobiodiversity provides a valuable resource for phytochemistry. This review presents an examination of essential phytochemicals found in plants and foods within Mexican agrobiodiversity that have shown promising anti-cancer and anti-diabetic properties, including their roles as antioxidants, insulin sensitizers, and enzyme inhibitors. Notable compounds identified include flavonoids (such as quercetin and catechins), phenolic acids (chlorogenic, gallic, and caffeic acids), methylxanthines (like theobromine), xanthones (such as mangiferin), capsaicinoids (capsaicin), organosulfur compounds (like alliin), and various lipids (avocatins). Although these phytochemicals have shown promise in laboratory and animal studies, there is a significant scarcity of clinical trial data involving humans, underscoring an important area for future research.

## 1. Introduction

Type 2 diabetes mellitus (T2DM) and cancer are increasingly prevalent non-communicable chronic diseases. According to the International Diabetes Federation, the prevalence of T2DM was 10.5% in 2021, with projections of 11.3% by 2030 and 12.2% by 2040 [1]. On the other hand, nearly 20 million new cancer cases were reported in 2022, resulting in 9.7 million deaths [2]. Prediabetes and T2DM are frequently observed among cancer patients, particularly following diagnosis [3], and T2DM is a significant cause of non-cancer mortality in this population [4]. Addressing the interconnected challenges posed by these conditions is essential for improving patient outcomes.

“Phytochemicals” are a diverse group of natural chemical compounds produced by plants responsible for various bioactive properties [5] and have garnered interest within the scientific community. Phytochemistry research has focused on two key areas: firstly, the diversity of bioactives present in plants and, secondly, the array of effector mechanisms they offer as adjuvant therapy to enhance conventional treatments for non-communicable chronic diseases such as cancer and T2DM. Thus, sufficient evidence has been reported confirming dietary phytochemicals’ anti-cancer [6] and anti-diabetic potential [7] based on their antioxidant/pro-oxidant properties.

The pursuit of phytochemicals has positioned Mexico advantageously, thanks to its esteemed international reputation for exceptional agrobiodiversity, which encompasses a wide array of agricultural products. This richness in agrobiodiversity provides access to various plants and plant-based foods that are sources of macro- and micronutrients, as well as a variety of phytochemicals with notable bioactive properties [8,9]. Recently, Barrera-Vázquez et al. [10] conducted a review using chemoinformatic screening to investigate the anti-aging, anti-inflammatory, and antioxidant properties of phytochemicals derived from Mexican medicinal plants. This manuscript’s novelty lies in its review of the anti-cancer and anti-diabetic potential of phytochemicals from representative plants and plant-based foods in Mexico’s agrobiodiversity. The paper also aims to review standard protocols for isolating phytochemicals and verifying their bioactivity in vitro and in vivo. Literature published in either Spanish or English from 2000 onwards, was gathered from three databases (Web of Science, Google Scholar, and PubMed).

## 2. Mexican Agrobiodiversity

Agrobiodiversity refers to the intersection of biological diversity and culture that shapes food production and human nutrition in a specific geographic region. Various factors, including ecosystems, agricultural production systems, social dynamics, cultivated plants, and conservation policies, influence it. In Mexico, agrobiodiversity provides access to a diverse array of plants and plant-based food sources that are rich in proteins, fats, minerals, vitamins, and energy, all of which are crucial for food security and human nutrition [8]. Additionally, Mexico’s ecosystems (including soil types, climates, and abiotic factors), along with genetic diversity, support the cultivation and consumption of plant-based foods that are high in phytochemicals with valuable bioactive properties [9].

**Figure 1:**
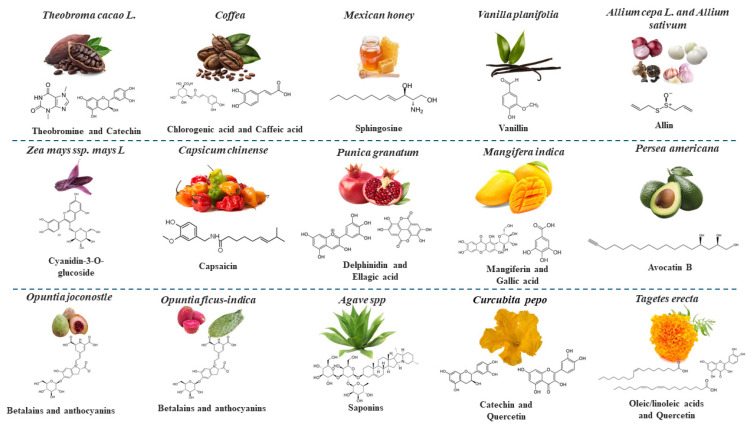
Mexican plants and plant-based foods representative of Mexican agrobiodiversity.

Species of Agave (*Agave* spp.), Vanilla (*Vanilla planifolia*), Coffee (*Coffea arabica* L. [from Chiapas]), Avocado (*Persea americana*), Cocoa (*Theobroma Cacao* L), Habanero chili (*Capsicum chinense* [from Yucatan]), Bee honey (*Apis mellifera* [from Yucatan]), Flor de Cempasúchil (*Tagetes erecta* [Nahuatl “Cempohualxochitl”]), Xoconostle (*Opuntia joconostle* [Nahuatl “Tuna amarga,” “xococ”]), Pomegranate (*Punica granatum* L.), and some varieties of Mango (*Mangifera indica* L. [Ataulfo]) are native to Mexico. On the other hand, species of Agave, Coffea (from Chiapas), Mango (Ataulfo), Vanilla (from Papantla), Habanero pepper (from Yucatan), and Cacoa (Grijalva) have a designation of origin [11]. Others such as Corn (*Zea mays* L.), Garlic (*Allium sativum* L.), Onion (*Allium cepa* L.), Nopal (*Opuntia ficus-indica*), and Pumpkin (*Cucurbita pepo* [and their flowers]) belong to the milpa (from the Nahuatl milpan from milli “planted plot” and pan “on top of”), a traditional polyculture agricultural system that favors a balanced diet and food sustainability based on the consumption of regional foods (Figure 1) [9,12].

## 3. Protocols for Phytochemical Screening

Table 1 outlines the protocols for phytochemical screening of plants and plant-based foods derived from Mexican agrobiodiversity. Firstly, the matrices undergo a drying process for extraction, which can be simple air drying at room temperature or more advanced techniques like freeze-drying and oven-drying. Once dried, the matrices are processed into purees, powders, or crushed forms to reduce particle size and enhance the efficiency of phytochemical extraction. Extraction of phytochemicals is typically performed using maceration at room temperature, either in light or dark conditions. It involves solvents with varying polarities, depending on the specific analyte to be extracted. In some cases, advanced technologies such as high-pressure homogenization [13], microwave irradiation, and ultrasonic irradiation are employed [14,15,16,17,18,19,20]. These methods facilitate mass transfer between the solvent and the extraction matrix, allowing for quicker recovery of phytochemicals.

Phytochemicals, such as polyphenols and flavonoids, are quantified using spectrophotometric methods. However, more sophisticated techniques, such as High-Performance Liquid Chromatography (HPLC) and Gas Chromatography/Mass Spectrometry (GC-MS) [21,22], are used for the specific identification and quantification of phytochemicals. It is important to note that some reports highlight the practical applications of the extracts, even when the phytoconstituents have not been definitively identified; thus, bioactive properties are often associated with crude extracts rich in phytochemicals.

## 4. Protocols for Bioactivities Determination

The anti-diabetic properties of phytochemicals have been widely confirmed through in vitro studies that show their ability to inhibit amylolytic enzymes (α-amylase and α-glucosidase) (see Table 2) [28,35,36,37,38,39,40,41,42,43,44,45,46,47]. Phytochemicals interact with amylolytic enzymes, reducing enzymatic activity and decreasing carbohydrate digestion and glucose release during the gastric and intestinal phases. In addition to in vitro studies, diabetic murine models are commonly used to investigate anti-diabetic properties further (see Table 2). These models often involve inducing diabetes in rodents using drugs that damage β-pancreatic cells, such as alloxan and streptozocin [48,49,50,51,52,53,54,55,56,57,58,59]. Many protocols also include administering high-fat and high-carbohydrate diets, such as fructose and sucrose [49,60,61,62,63]. The administration of phytochemicals during or after the onset of diabetes has demonstrated positive effects on metabolic responses (including insulin and lipid levels) and essential tissues like the pancreas and liver. Phytochemicals can enhance insulin sensitization, facilitate glucose transporter translocation, and improve glucose absorption in muscle tissue. However, it is essential to note that few studies have validated the anti-diabetic properties of phytochemicals in human clinical trials [64,65,66,67].

The anti-cancer properties of various phytochemicals have primarily been confirmed through in vitro studies on several cancer cell lines, including those for breast, prostate, gastric, colon, lung, pancreatic, ovarian, cervical cancers, and leukemia (see Table 3). There is substantial documentation on the modulation of biomarkers (such as genes and cytokines) related to different stages of carcinogenesis. Additionally, research has shown a reduction in tumor incidence in studies involving dogs and xenograft murine models. However, limited evidence exists regarding the anti-cancer effects of these phytochemicals in humans [68] and rodent studies [69,70,71,72,73].

**Table 2:** Anti-diabetic mechanisms and common experimental models.

Scientific Name	Source	Identified Phytochemical	Experiment Model	Anti-diabetic Mechanism^a^	Reference
*Theobroma Cacao* L.	--	-	α-glucosidase inhibitory assay	↑ Inhibition of α-glucosidase enzyme	[41]
		Theobromine and procyanidin B	3T3-L1 cells	↑ Phosphorylation of INSR tyrosine, PKC, and P13K proteins ↑ GLUT 4 translocation ↑ Glucose uptake	[74]
	Cocoa liquor	Procyanidins	Disease mice	↑ AMPK activation ↑ GLP-1 ↑ GLUT 4 translocation	[75]
	Cocoa bean (raw and roasted)	Flavan-3-ols, procyanidins, and flavonoids	Human hepatoma HepG2 and mouse insulinoma β-TC3 cells/Wistar rats (high-fat and low-fiber diet)	↓ Intracellular oxidative stress and ROS ↑ Glutathione reductase	[76]
*Persea americana* Mill	Fruit and leaves	-	α-amylase inhibitory assay	↑ Inhibition of α-amylase	[36]
	Leaves	-	α-glucosidase inhibitory assay (enzymes extracted from rats)	↑ Inhibition of α-glucosidase	[77]
	Seeds	-	Alloxan-induced diabetic rats	↑ Insulin and c-peptide secretion ↑ Beta-cell regeneration and function ↓ Insulin resistance	[78]
	Leaves	Hentriacontane -1-methylheptadecyl-benzene -1-cyclohexylethyl-benzene -Decyl heptyl ether, -Carbonic acid decyl tetradecyl ester -3,8-dimethyl-decane -Carbonic acid octa-decyl vinyl ester -2,6,10,15-tetramethyl-heptadecane -2,4-di-tert-butylphenol -3,5-bis (1,1 dimethylethyl) phenol]	α-glucosidase and α-amylase inhibitory assay (enzymes extracted from rats)	↑ Inhibition α-glucosidase, maltase-glucoamylase, aldose reductase, and aldehyde reductase activities	[45]
*Mangifera indica* L.	Several mango varietals	Mangiferin	Diabetic mice and albino rats	↓ Inflammation/oxidative stress, ↑ insulin signaling/glycolipid metabolism/	[79]
	Mango Ataulfo peel	Gallic acid + mangiferin rich extract	Streptozotocin-induced diabetic Wistar rats	↓ Fasting blood glucose/lipids), ↑ insulin sensitivity (HOMA-IR, HOMA-β)	[80]
	Mango leaves	PGG (EA-7/8-9/10-4 fraction)	High-fat-induced diabetic C5BL/6 mice	11β-HSD-1 inhibition, ↓adipogenesis	[63]
*Zea mays* L.	Several Peruvian corns	Free anthocyanins > phenolic acids	In vitro enzyme inhibition	↓ α-glucosidase and α-amylase activity	[43]
	Purple corn	Cyanidin-3-0 glucosides	Dual-layer cell cultures (Caco-2 (intestinal) cells, INS-1E (pancreas) or HepG2 (hepatic) cells)	↑ Insulin secretion/↓ epithelial glucose uptake/	[81]
	Blue/yellow corn	Cyanidin-3-0 glucosides	α-glucosidase and α-amylase inhibitory assay	↓ α-amylase activity	[40]
*Agave americana*	Agave fresh leaves	Phenolic acids/flavonoids	Alloxan-induced diabetic Wistar rats	↓ Hyperglycemia (days 15–45) and oxidative stress (liver, brain)	[52]
	Agave sap (“Aguamiel”)	Saponins	HF-induced obese C57BL/6 mice	↓ Hyperglycemia	[82]
*Coffea canephora*			Type 2 diabetes patients (*n* = 52)		
	Coffee	Chlorogenic acid	Streptozotocin-high fat diet-induced diabetic rats	-	[64]
*Coffea arabica*	Coffee	-	Streptozotocin-high fat diet-induced diabetic rats	↓ Nephrotoxicity, antioxidants with anti-inflammatory	[62]
	Coffee	-	High-fat diet-induced diabetic obesity in mice	Antioxidant, protection of liver oxidative damage	[49]
	Coffee	Chlorogenic acid	Alloxan-induced diabetic mice	↑ 5′-AMP-activated protein kinase (AMPK) activity	[60]
*Vanilla planifolia*	Vanilla	-	Streptozotocin-fructose induced diabetic rats	-	[83]
	Vanilla	Vanillin	Streptozotocin-induced diabetes rats	Improve metabolic parameters ↑ Pancreatic β-cell function ↑ glucose tolerance	[48]
	Vanilla	Vanillin	Streptozotocin-induced diabetic rats	↓ Renal expression of NF-κB and concentrations of IL-6, TGFβ1 and collagen. ↓ serum AGEs concentration	[84]
*Opuntia ficus-indica*	Prickly pear	-	Rat L6 myoblasts	↓ Post-pandrial hyperglycemia by decreased intestinal glucose absorption	[85]
	Nopal	-		↓ α-glucosidase activity. ↑ Peripheral Glucose Uptake through Activation of AMPK/p38 MAPK Pathway	[86]
*Capsicum chinense*	Habanero pepper	Capsaicin	Docking molecular	↓ α-amylase and α-glucosidase activity	[46]
	Habanero pepper	Capsaicin	α-amylase and α-Glucosidase inhibitory assay	↓ α-amylase and α-glucosidase activity	[37]
	Habanero pepper	Capsaicin	Clinical intervention study, 30 subjects aged 25–50, 7-day intervention period with 8 g of chili product	-	[67]
*Apis mellifera*	Bee honey	Flavonoids	α-amylase and α-glucosidase inhibitory assay	↓ α-amylase and α-glucosidase activity	[28]
	Bee honey	-	Streptozotocin-induced diabetes mice	↑ SOD GSH Y CAT	[50]
	Bee honey	Polysaccharides	Streptozotocin-induced diabetes mice (C57BL/6)	↓ Blood lipid reduction ↓ Oxidative stress resistance	[51]
	Bee honey	-	Streptozotocin administration plus high-fat and high-sugar diet-induced diabetes rats	↓ Dyslipidemia ↓ Oxidative stress	[61]
*Opuntia joconostle* Web	Xoconostle	-	Clinical trial in individuals with DM2	↑ Insulin levels	[87]
		-	Streptozotocin-induced diabetes in rats	-	[88]
*Opuntia matudae* Scheivar	Xoconostle	Betalains Isorhamnetin-3-O-rutinoside	Streptozotocin (STZ)-induced diabetic mice/Hepatic glucose output (HGO) model	-	[29]
		Chlorogenic acid, rutin, Quercetin-3-O-β-glucopyranose		-	
*Punica granatum*	Pomegranate	-	Streptozotocin (STZ)-induced diabetic rats	-	[53]
		-	Alloxan-induced diabetic mice	↑ Insulin levels	[59]
		-	Alloxan-induced diabetic rats	↑ Insulin levels	[57]
		-	Single-blind randomized clinical trial	↓ Oxidative stress	[89]
		Punicalagin, pyrogallol, ellagic acid, p-hydroxybenzoic, catechol, catechin, gallic acid, hesperidine, quercetin, kaemp-3-(2-p-comaroyl) glucose, naringin, apig-6-rhamnose 8-galactose, luteo-7-glucose, and hespertin	Alloxan-induced diabetic rats	↓ Oxidative stress	[56]
		Punicic acid	Randomized clinical trial	↑ Glucose Transporter type 4 (GLUT4) gene expression	[66]
		Rutin, gallic acid, nictoflorin, and tulipanin	Streptozotocin-nicotinamide (STZ-NA)-induced diabetic rats	↑ Insulin levels	[54]
*Allium cepa*	Purple onion (*Allium cepa* var. cepa)	Allicin constituents protocatechuic acid, vanillic acid, p-hydroxybenzoic acid, ferulic acid, and phloroglucinol	In vitro approach	Inhibitory effect on angiotensin I–converting enzyme Hydroxyl radical (OH ^·^) scavenging	[44]
	White onion (*Allium cepa* var. viviparum)	Allicin constituents Protocatechuic acid, vanillic acid, p-hydroxybenzoic acid, ferulic acid, and sinapinic acid	α-amylase inhibitory assay	Inhibitory effect on α-amylase activity -	[44]
	Onion peel	Quercetin	Streptozotocin (STZ)-induced diabetic rats	↑ Glucose tolerance ↑ Insulin-sensitizing effect ↑ Expression of insulin receptor ↓ IL-6 and oxidative stress	[58]
	Peels and bulbs		α-amylase and α-glucosidase inhibitory assay	Inhibitory effect on α-amylase and α-glucosidase activity	[38]
	Shallot (*Allium ascalonicum* L.)	-	Alloxan-treated rats	Improve the histopathological feature of rats’ liver induced by alloxan	[55]
	Brown onion	Quercetin	α-amylase inhibitory assay	Dose-dependent α-amylase inhibition	[90]
*Allium sativum*	Garlic	Allicin constituents p-coumaric acid, p-hydroxybenzoic acid, caffeic acid, ferulic acid, and sinapinic acid	α-Glucosidase inhibitory assay	Inhibitory effect on α-glucosidase activity Higher Fe2+ chelating ability	[44]
	Garlic and onion oils	-	In vitro approach	Inhibitory effect on α-glucosidase	[42]
	Aged garlic	-	Double-blinded placebo-controlled, randomized clinical trial (n = 65) on type 2 diabetes patients	↓ Cardio-ankle vascular index	[65]
*Cucurbita pepo* L.	Squash flower	(+)-catechin, (-)-epicatechin, rutin, syringic acid, hesperidin and quercetin 3-O-glucoside	α-Glucosidase inhibitory assay/Hypoglycemic assay in healthy mice	↓ α-glucosidase activity	[39]
*Tagetes erecta*	Cempasúchil	Quercetagetin, 6-hydroxykaempferol -3-O-hexoside, patuletin-7-O-hexoside	α-Glucosidase inhibitory assay	↓ α-glucosidase activity	[47]
		Quercetin, hyperoside, isoquercitrin, ellagic acid, and vanillic acid	α-Glucosidase inhibitory assay	↓ α-glucosidase activity	[35]

^a^ ↑: increase; ↓: decrease.

**Table 3:** Anti-cancer mechanisms and common experimental models.

Scientific Name	Source	Identified Phytochemical	Anti-cancer Mechanism^a^	Experiment Model	Reference
*Theobroma Cacao* L.	Cocoa leaves	39 bioactive compounds	↑Apoptosis ↑Caspase-3, -8, and -9 ↓BAX, BNIP3 ↑Pro-apoptotic genes (DDIT3, GADD45G, and HRK). Disruption of mitochondrial membrane potential	Human breast cancer cell line (MCF-7)	[91]
			↑Doxorubicin-induced oxidative stress	A549, Ehrlich’s ascites carcinoma, Chinese Hamster Ovary cancer cell lines	[92]
			↓Doxorubicin-induced organ (heart, liver, and kidney) toxicities	Mice	[93]
	Cocoa husk	Catechin (5.64 mg/g), epicatechin (20.47 mg/g), and procyanidin (20.29 mg/g)	↑Apoptosis ↑DNA fragmentation	PC3 and DU145 prostate cancer cells	[94]
*Persea americana*	Leaves	Chlorogenic acid, naringenin, rutin, ferulic acid, syringic acid, gallic acid, coumaric acid, caffeic acid, methyl gallate, vanillin, and ellagic acid	↑Anti-cancer activity (IC50 = 44.28–90.52 μg/mL)	Human lung normal fibroblast (Wi38) and human lung carcinoma (A549) in addition to human liver carcinoma (HepG2) cell lines	[95]
	Leaves	Flavonoids Tannin Quinone Steroid	↓ Cell cycle in the G1/S and G2/M phases	Human squamous carcinoma (HSC-3) cell lines	[96]
	Pulp and seeds	5-Hydroxymethylfurfural, 9-Octadecyne, and 9-octadecenamide	↓ Tumor markers (AFP, CEA, CA19.9) ↑ LPO and GSH ↑ SOD, GST, GPx ↓ p53, COX-2, and NF-κB mRNA expressions	Male adult Wistar rats with induced hepatocarcinogenesis	[97]
	Seeds	Avocatins B Polyhydroxylated fatty alcohols Long-chain fatty acids	Cytotoxic effect (IC50 = 28 μg/mL) ↑ Apoptosis ↑ Caspases 8 and 9 ↑ IL-6, IL-8, and IL-10 ↓ IL-1β ↓ Mitochondrial membrane potential	Caco-2 human colon cancer cells	[22]
*Mangifera indica* L.	Mango (“*Ataulfo*”) pulp	Gallic acid, gallotannins	↓ Cell viability, ↑ apoptosis	Cervical (HeLa) cancer cells	[19]
.	Mango (“*Ataulfo*”) peel	Gallic acid, gallotannins	↓ Cell viability, ↑ apoptosis	Colon (LS180) cancer cells	[18]
	Mango (“*Keitt*”) pulp	Gallic acid, gallotannins	↓ Cell viability, ↑ apoptosis	TNF-α treated MCF-12A (normal) and MDA-MB231 (cancer) breast cells	[98]
*Zea mays* L.	Blue corn and tortilla	Cyanidin-3-*O*-acyl/alkyl-glucosides	G1-cell cycle arrest, ↑ apoptosis	Androgen-sensitive breast/prostate cancer cells	[99]
	Corn silk	Maysin. luteolin	↓Cell viability, ↑ apoptosis (ROS-mediated)	Breast cancer (MCF-7) cells	[100]
	Purple corn	Cyanidin-3-0 glucosides	↓ DMBA-induced mammary tumor development, ↑ apoptosis (↑ Caspase-3, ↓ Ras protein)	Cancer-prone transgenic (Hras128) mice	[101]
*Agave* spp.	Agave sap (“*Aguamiel*”)	Magueyoside B, gentrogenin tetraglycosides	↓ Cell viability (dose-dependent)	Colon (Caco-2) cancer cells	[102]
*Agave salmiana*	Dry fructan mixtures	Highly-branched fructans	↓ Cell viability/proliferation, ↑ apoptosis	Colon (HT-29/SW480) cancer and healthy (CRL1831) cells	[103]
*Coffea arabica*	Coffee	Chlorogenic acid	↓ Cell viability and cell cycle arrest in S and G2/M ↑Induced apoptosis	Prostate adenocarcinoma cell lines (PC-3 and DU-145)	[104]
	Coffee	Chlorogenic and gallic acids	↑ Pro-apoptotic mechanism inducing cell toxicity by increased Caspase-3 activity and mitochondria dysfunction	Human colon adenocarcinoma SW480	[105]
*Coffea canephora*	Coffee	____	Induction of S phase and ↓ G2/M population in breast cancer cells affected the mitochondrial morphology and triggered apoptosis	MDA-MB-231 (ER-) and MCF-7 (ER+) breast cancer cells	[106]
	Coffee	Chlorogenic acid	↓ Transcriptional activity of β-catenin	SW480 and HT-29 CRC cells	[107]
	Coffee	Chlorogenic acid	↓ Inflammatory cytokines TNFα and IL-6	Colorectal cancer induced in Wistar rats	{69]
*Vanilla planifolia*	Vanilla	Vanillin	↑ Apoptosis by cytochrome c and caspase-9 upregulation.	Human colorectal adenocarcinoma cell line (HT-29)	[108]
	Vanilla	Vanillin oxime	Inhibition of pulmonary cell proliferation, ↑ apoptosis through tumor necrosis factor-related apoptosis-inducing ligand (TRAIL) mediated pathway	Lung cancer cells (A549 and NCI-H2170)	[109]
*Opuntia ficus-indica*	Prickly pear	17-hydroxy betanin	___	Ehrlich ascites carcinoma cells (EACC)	[110]
	Mexican prickly pear (n = 9)	Phenolic acids, flavonoids, betaxanthins, and betacyanins	↓ Cell viability	Breast (MCF-7), prostate (PC3), colon (Caco2), and hepatic (HepG2) cell lines	[111]
	Stem	_____	Decreased cell viability ↓ Cyclooxygenase-2 and ↑ Bax/Bcl2 ratio	Human SW480 colon and MCF7 breast cancer cells	[112]
	Prickly pear	Indicaxanthin	↓ NF-κB pathway	Human melanoma cells	[113]
*Capsicum chinense*	Habanero white	Quercetin derivatives	↓ Inflammation ↓ ROS	Human monoblastic leukemia U937	[114]
	Habanero pepper	Polyphenols	___	Parental breast cancer cells, MCF-7/Sens	[22]
	Habanero pepper	Capsaicin	___	D-17 and DAN cells	[27]
	Habanero pepper	Capsaicin	Inhibition affects the electron transport from NADH to ubiquinone, binding of pasaicin with co-enzyme Q	In vivo, 53 dogs aged/-15 years with hepatic tumors	[68]
	Chile habanero	Capsaicinoid	↑ Level of expression of proapoptotic ↑ Activity of liver enzymes	Swiss albino mice Induction cancer with DMBA/Human hepatocellular carcinoma cell lines (Hep 3B and Hep G2)	[73]
*Apis mellifera*	Bee honey	___	___	Human breast cancer (MCF-7) cells and human cervical adenocarcinoma (Hela) cells	[115]
	Bee honey	Sphingoid, phytodphingosine, sphinganine	___	Cyclophosphamide-treated human lung carcinoma (A549) cells	[116]
	Bee honey	Polyphenols	Inhibition of lipoprotein oxidation, induction of apoptosis pathway	Human breast cancer cell line MDA-MB-231	[117]
	Bee honey	Honey sugars analogs	↑ Susceptibility of expression of proapoptotic proteins such as Apaf-1, caspase-9, IFN-c, INF-c, IFNGR1 ↓ Expression of antiapoptotic proteins such as E2	Female rats, model breast cancer induced by 1-methyl-1-nitrosourea	[118]
	Bee honey	Caffeic acid, ferulic acid, mannose, aloin A, aloin B, pino cembrin, chrysin and kaempfero	↓ Metastatic propensity of tumors by modulating neoangiogenesis and the process of epithelial to mesenchymal transition	Male Wistar rats, with subcutaneous tumor implants	[71]
*Opuntia joconostle*	Xoconostle	--	Arrest of cells in the G2/M phase of the cell cycle	Breast cancer cell lines MCF-7 and MDA-MB-231	[119]
*Punica granatum*		--	↓ Metastasis Upregulation of E-cadherin and ICAM-1 genes Downregulation of HMMR and Type I collagen genes Upregulation of anti-invasive miR-335, miR-205, miR-200, and miR-126 Downregulation of pro-invasive miR-21 and miR-373 ↓ Levels of IL-6, IL-12p40, IL-1β, and RANTES.	Human prostate cancer epithelial cell lines DU145, LNCaP, and PC-3	[47]
		Punicalin, punicalagin, and ellagic acid	Induction of apoptosis through mitochondrial impairment	Human oral cancer cell lines Ca9-22, HSC-3, and OC-2	[120]
		--	--	Breast ductal carcinoma cell lines T-47D and HTB-133 TM/Breast cell lines MDA-MB-231 and HTB-26 TM/Alveolar cell lines A549 and CCL-185 TM/colorectal Duke’s type II adenocarcinoma cell lines LS180 and CL-187 TM	[31]
		Gallic acid, 3-hydroxytyrosol, vanillic acid, rosmarinic acid, 4-hydroxybenzoic acid, chlorogenic acid, caffeic acid, syringic acid, p-coumaric acid, resveratrol, salicylic acid, quercetine-3-Oglucoside	Induction of apoptosis ↓ Reduction in oxidative stress	Human breast cancer cell line AU565-PAR	[121]
*Tagetes erecta*	Cempasúchil	Lacritirin	Induction of apoptosis	Human ovarian carcinoma cell line A2780	[122]
		2,3-dihydro benzofuran (coumaran), octadecanoic acid, benzene acetic acid, oleic acid, linoleic acid, acetic acid, methyl-D-galactopyranoside, n-butyric acid, methyl-L-arabinopyranoside, and n-hexadecanoic acid	↓ Mitoses	Lewis lung carcinoma cell line LLC1/Human breast carcinoma cell line MCF-7/Xenograft lung carcinoma model	[72]
		Limonene, cis-ocimene, (+)-2-carene, sabinene, carene < delta- 3- >, dihydrotagetone, Artemesia ketone, neo-allo-ocimene, myroxide, ocim-(4E,6Z)-ene < allo- >, tagetone < (Z)- >, piperitone	Anti-apoptotic effect/anti-inflammatory response through downregulation of Nrf2/HO-1 and NF-κB signaling pathways	N-methyl-Nʹnitro-N-nitroguanidine (MNNG) induced gastric cancer in rats	[70]
*Allim cepa*	Onion bulbs	Quercetin and quercetin-glycosides	↑ Toxicity on cancer cells ↑ G2/M phases ↓ G1 phase	Adrenocortical carcinoma cell line (H295R and SW-13)	[123]
	Onion peel	–	↓ Viability Apoptosis ↓ Migration and invasion ↓ L1CAM and NF-κB ↓ Angiogenesis	HT-29 cells	[124]
	Welsh onion leaves	Allicin Allin Isoquercitrin, ferulic acid, rutin	Dose-dependent antiproliferative potential ↑ Casp3 ↑ Catalase ↑ Lactate dehydrogenase	Human normal fibroblasts (BJ) and human normal keratinocytes (HaCaT)	[125]
	Red onion	Quercetin	Apoptosis ↓ Cell proliferation ↑ CYP1A1 and CYP1B1 gene expression	MDA-MB-231 cells	[126]
*Allium sativum*	Garlic bulbs	Allicin Allin Chlorogenic acid p-coumaric acid 4-hydroxybenzoic acid.	Dose-dependent antiproliferative potential ↑ Casp3 ↑ Lactate dehydrogenase	Human normal fibroblasts (BJ) and human normal keratinocytes (HaCaT)	[125]
	Aged garlic	S-allyl-L-cysteine	Anti-proliferative effects in a dose-dependent manner ↑ Apoptosis ↓ ΔΨm (mitochondrial membrane depolarization) ↑ oxidation of endogenous glutathione	Neuroblastoma cancer cells	[127]
	Black garlic	–	Apoptosis ↓ Anti-apoptotic protein MCL-1 ↓ BCL-2 ↑ BAX and BIM	MCF-7 and MDA-MB-361 breast cancer cells	[128]
	Purple garlic	Thiosulfinate	↓ Cell viability (≥70% [dose–response]) ↓Cell integrity increase ↑5-FU/oxaliplatin efficacy	Caco-2 and HT-29 cells	[129]
	Garlic	–	↓ Cell viability and cell proliferation (dose-dependent (0–25 μM))	A549 and H1299 lung cancer cells	[130]

^a^ ↑: increase; ↓: decrease.

**Figure 2:**
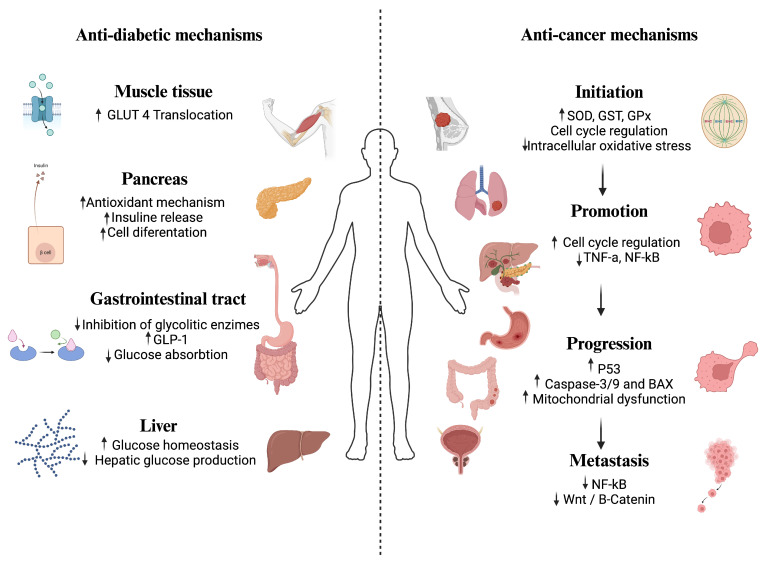
Mechanisms of anti-diabetic and anti-cancer effects of Mexican plants and plant-based foods. The anti-diabetic mechanisms of phytochemicals are primarily based on their ability to inhibit glycolytic enzymes and reduce glucose absorption in the intestines. They also promote the secretion of glucagon-like peptide-1 (GLP-1). At the pancreatic level, these compounds decrease oxidative stress and support the proper differentiation of β-cells while enhancing insulin secretion. Additionally, phytochemicals facilitate the translocation of glucose transporters (GLUT4) in muscle tissue, aiding in the cellular uptake of glucose. They also promote the correct metabolism and homeostasis of glucose in the liver. In terms of cancer, phytochemicals positively influence various stages of the disease. During the initiation phase, they regulate the cell cycle and improve the antioxidant environment by increasing levels of antioxidant enzymes such as superoxide dismutase (SOD), glutathione S-transferase (GST), and glutathione peroxidase (GPx). In the promotion phase, phytochemicals downregulate the production of cytokines associated with tumor progression, precisely tumor necrosis factor (TNF-α) and nuclear factor kappa light-chain enhancer of activated B cells (NF-κB). During the progression phase, they activate pro-apoptotic genes (like P53) and pro-apoptotic proteins (Caspase-3/9 and BAX). Finally, in the metastasis phase, phytochemicals reduce the activity of NF-κB and Wnt/B-catenin pathways.

The anti-diabetic and anti-cancer properties of phytochemical-rich extracts from plants and plant-based foods, representing Mexican agrobiodiversity, are discussed further below and illustrated in Figure 2.

## 5. Cocoa (*Theobroma cacao* L.)

Cocoa (*Theobroma cacao* L.) is a seed-type fruit that consists of pods containing beans and a sweet, mucilaginous pulp (cotyledon, 20%), all surrounded by a husk or bark (80%). The market size for cocoa is estimated to be USD 17.24 billion in 2024 and is projected to reach USD 23.97 billion by 2029. Cocoa is native to the rainforest regions of tropical America and is currently cultivated primarily in the tropics of America, Africa, and Asia. In Mexico, cocoa cultivation is mainly concentrated in the southeastern part of the country, with evidence of its cultivation dating back to 1900 BC in pre-colonial Mesoamerica. In Chiapas, near the border with Guatemala, the Criollo variety (*Theobroma cacao* subsp. cacao) is produced and accounts for 30% of the national crop [20]. Cocoa seeds and pulp are valued for their flavor, aroma, and health benefits, which include anti-diabetic and anti-cancer properties.

### 5.1. Anti-Cancer Properties

Cocoa by-products, including pods, beans, and leaves, are significant sources of phytochemical-rich extracts that contain various bioactive compounds. These include flavanols, procyanidins, stilbenes, flavonoids, methylxanthines, alkaloids, tannins, and saponins [131]. Cocoa phytochemicals exhibit anti-cancer properties through different mechanisms, demonstrated in vitro, in silico, and in vivo (see Table 3).

In a recent study, Ranneh et al. [91] showed that cocoa leaf extract induces apoptosis in MCF-7 cells by upregulating pro-apoptotic genes and activating multiple pathways, including the death receptor pathway (Caspase-8), mitochondrial pathway (Caspase-9), and effector caspase pathway (Caspase-3). Similarly, cocoa husk extract—rich in catechin (5.64 mg/g), epicatechin (20.47 mg/g), and procyanidin (20.29 mg/g)—demonstrated concentration-dependent anti-cancer effects through apoptosis in PC3 and DU145 prostate cancer cells [94]. Furthermore, cocoa extract has shown a synergistic effect with current oncological drugs, enhancing the efficacy of doxorubicin in inhibiting cancer cell proliferation while reducing doxorubicin-induced organ toxicity. Therefore, cocoa can be a nutraceutical or complementary medicine [92,93].

### 5.2. Anti-Diabetic Properties

Cocoa by-products exhibit anti-diabetic properties by regulating glucose homeostasis and modulating insulin signaling pathways. They also demonstrate antioxidant mechanisms on β-cells and inhibit carbohydrate digestive enzymes (see Table 3). Theobromine (a type of methylxanthine) and procyanidin B1 counteract the inhibition of insulin signal transduction by promoting the phosphorylation of key proteins such as INSR tyrosine, PKC, and PI3K.

Additionally, these compounds enhance the translocation of GLUT4 to the cell membrane (by 59.8% to 79.3%) and stimulate glucose uptake (by 75.4% to 80.4%) [74]. A procyanidin-rich extract and its fractions—low DP (degree of polymerization ≤ 3) and high DP (degree of polymerization ≥ 4)—obtained from cocoa liquor help prevent postprandial hyperglycemia. This effect is accompanied by increased glucagon-like peptide-1 (GLP-1) and insulin plasma levels, activating its downstream signaling pathway in skeletal muscles, which results in GLUT4 translocation [75].

Moreover, cocoa bean extracts reduce intracellular oxidative stress and the production of reactive oxygen species (ROS), which are associated with hyperglycemia and the dysfunction of antioxidant defense mechanisms [76]. Indrianingsih et al. [41] reported that extracts from cocoa pod husks (both purple and yellow variants) and their fractions (hexane, methanol, and dichloromethane) exhibited the highest α-glucosidase inhibitory activity, with IC50 values ranging from 10.8 to 41.6 μg/mL.

## 6. Coffee (*Coffea arabica* L. and *C. canephora* R.)

Coffee (*Coffea arabica* L. and *Coffea canephora*) is a globally significant commodity, ranking among the most traded and consumed beverages after water. In recent years, the coffee market has seen notable growth, with projections estimating a rise from USD 24.55 billion in 2023 to USD 26.2 billion in 2024 and further increasing to USD 33.09 billion by 2028. Mexico currently ranks fifth in global coffee supply, holding a market share of 3.5%. The value of the Mexican coffee market reached USD 2.15 billion in 2023 and is projected to grow to USD 3.53 billion by 2032 [132]. Coffee cultivation occurs in 12 states nationwide, with Chiapas accounting for 74% of the overall production [133]. The primary species cultivated in Mexico are *C. arabica* and *C. canephora*.

Coffee contains several bioactive components, including phenolic compounds (such as chlorogenic acids, cafestol, and kahweol), alkaloids (like trigonelline), and diterpenes (cafestol and kahweol) [134]. These phytoconstituents contribute to the prevention of diseases related to inflammation and oxidative stress, such as obesity, metabolic syndrome, T2DM, and cancer. Most studies associate chlorogenic acid specifically with coffee’s anti-diabetic and anti-cancer properties.

### 6.1. Anti-Cancer Properties

The anti-cancer potential of coffee and its derivates has been corroborated in several cancer cell lines or cancer-induced rat models [69]. Chlorogenic acid is the main phytoconstituent explored for prostate, colon, and breast cancer cell lines [104,106,107]. Documented mechanisms include the induction of apoptosis, mitochondrial dysfunction, and downregulation of tumor progression-related cytokines (TNFα, NF-κB, and IL-6), as well as upregulation of pro-apoptotic proteins (caspase-3) (Table 3).

### 6.2. Anti-Diabetic Properties

The anti-diabetic properties of coffee extracts from *Coffea arabica* or *Coffea canephora* have been studied using diabetic–obese murine models induced by a high-fat diet and/or streptozotocin. However, there is limited experimental evidence from clinical trials involving human populations [64]. Most of the studies focus on aqueous extracts from green coffee, although some evidence supports roasted coffee extracts. The phytoconstituents most explored include caffeine, caffeic acid, and chlorogenic acid.

Among the reported beneficial effects is lowering blood glucose levels, likely due to the upregulation of 5′-AMP-activated protein kinase (AMPK) (Bashir et al., 2023). Additionally, coffee extracts may help prevent damage to the kidneys, liver, or pancreas through their antioxidant properties [49,62] (Table 2).

## 7. Avocado (*Persea americana* Mill)

*Persea americana Mill* (also known as avocado, alligator, or ahuacate) is a fruit native to Mexico (var. dry mifolia) that belongs to the Lauraceae family and comprises approximately 50 species. Anatomically, the avocado fruit is pear-shaped with a tough outer skin, creamy pulp, and a large seed in the center (Figure 1). The seed and skin often generate large quantities of waste and by-products, which can be re-utilized for nutraceutical purposes. The Avocado market is estimated at USD 22.69 billion in 2024 and is expected to reach USD 35.55 billion by 2029. Mexico is the top avocado-producing country in the world, with an area of ~224,422 acres, producing over 2.3 million tons in 2020. Avocado Hass is the main Mexican variety (60%) for national intake and exportation. Other leading avocado producers include tropical countries such as Colombia, Peru, the Dominican Republic, and Indonesia [135]. In Mexican folk medicine, avocado fruits and by-products have been utilized as a source of bioactive components, including monounsaturated fatty acids, tocopherols (α-tocopherol, β-tocopherol, and γ-tocopherol), persin, persealide, proanthocyanins, and others [135].

### 7.1. Anti-Cancer Properties

The anti-cancer properties of avocado and its by-products have been extensively studied through various in vitro assays on different cell types. Avocado seeds, leaves, and pulp have been identified as potential sources of phytochemicals with anti-cancer effects (see Table 3). A notable study by Lara-Marquez et al. [22] examined the cytotoxic effects of a lipid-rich extract from the Mexican native avocado seed (*Persea americana* var. drymifolia) on Caco-2 colon cancer cells. The main bioactive components in this extract were avocatins and polyhydroxylated fatty alcohols, comprising 29.2% and 21.9%, respectively. The proposed anti-cancer mechanism involved increased production of reactive oxygen species (ROS), loss of mitochondrial membrane potential, induction of caspase-dependent apoptosis, and modulation of cytokines related to the inflammatory response.

Additional studies have shown the anti-cancer effects of avocado leaf extracts on lung carcinoma cells and liver carcinoma (HepG2) cells (Thessalonica and Roeslan, 2020). Furthermore, an in vivo study demonstrated that hydroethanolic extracts of avocado fruits and seeds improved the activity of antioxidant enzymes (SOD, GST, and GPx) while reducing tumor markers and inflammation mediated by COX-2 and NF-κB in rats with hepatocarcinoma [97].

### 7.2. Anti-Diabetic Properties

Various studies have supported the anti-diabetic properties of avocado fruits and their by-products, including seeds, leaves, and peels. A methanolic extract of avocado leaves exhibited potent inhibition of the α-glucosidase enzyme, showing dose-dependent effects (Ki = 1.4 mg/mL) [77]. Interestingly, α-amylase inhibition was higher in fruit extracts (92.13%) compared to leaves (88.95%) [36]. Additionally, avocado peel extracts have demonstrated anti-diabetic potential, with benzene aromatic compounds likely being significant inhibitors of glucosidases and maltase-glucoamylase [45].

## 8. Vanilla (*Vanilla planifolia*)

Vanilla (*Vanilla planifolia*) is a tropical plant native to Mexico, specifically from Oaxaca and Veracruz. This plant, belonging to the Orchidaceae family, consists of stems, orchids, and fruits, commonly called beans. Vanilla has been used for flavoring since the Totonac and Aztec cultures. After Hernán Cortés’s conquest, *Vanilla planifolia* was sent to Europe; however, cultivation there proved unsuccessful due to unsuitable climate conditions. It was later transferred to Réunion Island and Madagascar, where it thrived, and subsequently to Indonesia, the Philippines, and Tahiti [136].

Although Mexico is recognized as the center of origin for the genetic diversity of vanilla, Madagascar is currently the leading producer. The two primary species contributing to the global market are *Vanilla planifolia* and *Vanilla tahitensis*. The annual production of vanilla is approximately 8000 tons, and the market size is valued at around USD 1.12 billion. The increasing demand for natural flavors and the growth of the food and beverage sector are the main drivers of the vanilla market [137,138].

In Mexico, the demand for vanilla has increased by 30.49% over the last decade, especially in the food, cosmetics, and pharmaceutical industries. In 2023, the total trade exchange for vanilla (including international purchases and sales) was USD 629,000. The primary commercial destinations for Mexican vanilla were the United States (USD 208,000), France (USD 73,800), Germany (USD 45,700), Japan (USD 9860), and the United Arab Emirates (USD 106) [139].

Vanilla extracts contain a mixture of approximately 200 compounds. The characteristic flavor and fragrance are primarily derived from vanillin, vanillic acid, p-hydroxybenzaldehyde, and p-hydroxybenzoic acid. Although commercial species exhibit qualitative similarities in their metabolic composition—such as the ability to produce vanillin—there are significant quantitative differences. While vanillin is predominantly used as a flavor and fragrance ingredient, it also possesses diverse bioactive properties.

### 8.1. Anti-Cancer Properties

The anti-cancer potential of vanilla has been demonstrated in various cancer cell lines. Ethanolic extracts of pure commercial vanilla effectively reduce cell viability and induce apoptosis in colon, lung, cervical, and glioblastoma cancers. Vanillin, the primary phytochemical studied for its anti-tumor properties, targets proteins such as Transient Receptor Potential Vanilloid Subtype 1 (TRPV1), as well as cytoplasmic peptides like Microtubule Affinity-Regulating Kinase 4 (MARK4), Human Calcium/Calmodulin-Dependent Protein Kinase IV (CAMK4), and Casein Kinase II Subunit (CK2) [140].

Research indicates that vanillin binds to CAMK4, which helps inhibit the proliferation of cancer cells. It may also induce apoptosis by targeting the CK2α subunit, known for its role as an anti-apoptotic protein. Additionally, vanillin has the potential to inhibit MARK4, a protein that is often overexpressed in various cancers, making it a promising target for future studies. Other mechanisms include the upregulation of caspase-9 and the tumor necrosis factor-related apoptosis-inducing ligand (TRAIL) [140].

### 8.2. Anti-Diabetic Properties

Only a limited number of studies evaluate the effects of *V. planifolia* bioactive compounds on type 2 diabetes mellitus (T2DM). Singletary [141] reviewed the existing literature and suggested that vanillin and vanillic acid may have anti-diabetic potential. These compounds are thought to improve metabolic parameters related to obesity and enhance insulin sensitivity and glucose tolerance in rodents, such as mice and rats, that are fed high glucose/lipid diets (see Table 2). A recent study by Salau et al. [48] documented that vanillin suppressed blood glucose levels, serum cholesterol, triglycerides, and low-density lipoprotein cholesterol (LDL-c). Additionally, it improved pancreatic β-cell function, glucose tolerance, and pancreatic morphology in diabetic rats induced by streptozotocin and fructose.

## 9. Mango (*Mangifera indica* L.)

Mango (*Mangifera indica* L.; Anacardiaceae) is a tropical/climacteric fruit cultivated in many countries and considered a natural functional food. More than a thousand varieties have been described, yet few of them are commercialized in the global mango market, valued in 2024 at nearly USD 68 million, and that will grow at a compound annual rate (CAGR) of 7.1% by 2028 [142]. India and China dominate this market, yet Mexico ranks 4th, and it is estimated that 1 out of 20 mangoes consumed worldwide are of Mexicans; Ataulfo and Manila (yellow varietals), Tommy-Atkins, Hayden, and Kent (red varieties) are the most important Mexican cultivars [143,144].

Mango pulp or flesh (mesocarp, 33–85% w.w-1 upon varietal) (Figure 1) is mainly consumed fresh, frozen, canned, concentrated, and dried products [143], besides its sensorial attractiveness, it is rich source of vitamins, organic acids, carbohydrates/pectin, amino acids, phenolic and volatile compounds, in a varietal-specific manner [144]. Moreover, the mango agroindustry produces a vast amount (20–65% w.w-1) of non-edible wastes (e.g., flowers, leaves, and barks) and by-products (e.g., rind, peels (skin; exocarp), seed (endocarp), and bagasse), with a much higher nutraceutical potential than the pulp and which have been proposed/used as ingredients or source of functional xenobiotics for the food, cosmetic, and pharmaceutical industries [145].

Functional phytochemicals in mango byproducts include but are not restricted to fatty acids (linoleic/oleic), carotenoids (carotenes (α,β,γ-carotenes), xanthophylls (lutein, auro/anthera/neo/viola/zeaxanthin)), complex polysaccharides (e.g., highly methoxylated pectin and cellulose), and simple (phenolic acids (gallic/ellagic/hydroxycinnamic), xanthones (mangiferin), flavonoids (quercetin))-to-complex (e.g., gallotannins) phenolic compounds [146].

Lastly, phytochemicals from mango pulp and by-products exhibit several health-promoting benefits such as antioxidant, anti-inflammatory, immunomodulatory, antimicrobial, anti-cancer (Table 3), and anti-diabetic (Table 2) effects.

### 9.1. Anti-Cancer Properties

Several mango phytochemicals have anti-cancer effects (Table 3). Preclinical studies (in vitro, ex vivo, and in vivo) have unveiled central anti-cancer mechanisms and molecular targets of *M. indica*-derived constituents, including anti-proliferative, cell cycle regulation, pro-apoptosis, antiangiogenic, antimetastatic (invasiveness/migration), antioxidant, and anti-inflammatory activities. However, the anti-cancer activity of a given mango phytochemical depends on (i) the cancer type and developmental stage and (ii) the quantitative structure–activity relationship (QSAR)—the relationship between such phytochemical and target carcinogenic molecules [145]. Gallic acid (free and/or gallotanin-derived) from mango pulp and by-products has antiproliferative, pro-apoptotic, anti-inflammatory effects (all of them related to improvements in cell redox status and intracellular signaling) on breast (MDA-MB231 [98]), cervical (HeLa [19]), and colon (LS180 [18]) cancer cells. Conversely, the main anti-cancer activities of mangiferin are anti-inflammatory, antioxidant, and pro-apoptotic (as gallic acid) on a much broader cancer-cell list and seem to have more substantial antiangiogenic effects and synergistic effects when combined with chemotherapeutic drugs [147].

### 9.2. Anti-Diabetic Properties

T2DM is a chronic non-communicable disease with a well-defined clinical course (stages: acute/chronic hyperglycemia—insulin resistance—diabetic complications) and well-known physiological/genetic/molecular disturbances. The QSAR of mango phytochemicals toward this disease seems to be disease-stage-dependent (Table 2). Therefore, it is not surprising to observe an increase in patent applications for specific compounds aimed to ameliorate quite specific clinical diabetic features, from which gallic acid, Mangiferin, and 1,2,3,4,6-penta-O-galloil-D-glucose (PGG, gallotanin) seem to be the most promising anti-diabetic bioactives [148]. For example, Wu et al. [79] performed a systematic review/metanalysis with a PICO approach (patients–intervention–comparison–outcome) on the anti-diabetic action of mangiferin in rodent models, attributed to its anti-inflammatory/antioxidant activity and improving glycolipid metabolism and insulin signaling. Preciado-Saldaña et al. [80] reported that a mango “Ataulfo” hydroalcoholic extract (rich in mangiferin and gallic acid) improves metabolic dysregulation (↓ fasting blood glucose/lipids, ↑ insulin sensitivity (HOMA-IR, HOMA-β)) in streptozotocin-induced (25 mg·kg^−1^, single dose) prediabetic rats. Mohan et al. [63] isolated (EA-7/8-9/10-4 fraction) and identified PGG in an ethyl-acetate mango leaf extract and tested its anti-diabetic potential in high-fat induced diabetic C5BL/6 mice observing its potent inhibitory activity against 11β-hydroxysteroid dehydrogenase type 1 (11β-HSD-1), adipogenic enzyme, and further supported by in vitro (enzyme inhibition) and in silico evidence. Lastly, given that mango pulp contains more sugar than its by-products [144,149], research on its anti-diabetic potential has been scarcer.

## 10. Corn (*Zea mays* L.)

Corn or maize (*Zea mays* ssp. Mays L.; Zea (Greek: sustaining life), Mays (Taino: life giver)) is an edible grass belonging to the Poaceae family, and it is one of the top three domesticated staple crops in Latin America. The corn germplasm determines the kernel’s color and programmed phytochemical production of each varietal, among other phenotypic features. However, many environmental factors (e.g., altitude, temperature, abiotic factors) and pre-harvest factors (e.g., maturation state, agricultural practices) may also affect corn’s phytochemical profile. While white-to-yellow varieties are considered staple foods (e.g., cooked corncob) or vehicles for macronutrient-delivering (e.g., corn flours) that help to fulfill the nutritional requirements of people living in low-income countries [25], orange-pink and red-purple varieties have gained greater recognition as functional foods or as source of nutraceuticals (e.g., phenolic compounds, carotenoids) with structure-specific bioactivity for both primary prevention and secondary control of many NCCD. Red–blue–purple corn varietals are mainly used to prepare traditional or processed functional foods [14].

The corn market size was valued at USD 297.99 billion in 2023. The market is anticipated to grow from USD 307.91 billion in 2024 to USD 410.02 billion by 2032 [150]. Mexico is the seventh largest producer of corn in the world, with cultivation in all 32 states of the country. In 2021, 7 million hectares were allocated for cultivation, and corn production was 27 million tons [151]. Mexico not only ranks seventh in worldwide corn production, but 14–22% of all (~300) Latin-American maize races are grown in Mexico, mainly for self-consumption in rural communities [25]. Although corn varieties’ phylogenetic relationship (chromosomal nodes) is narrow, the agroecological performance and nutraceutical/functional value of each one are determined by its intrinsic metabolic activity. Several varietal-specific phytochemicals have been identified in corn kernels, and some of them have been considered responsible for health benefits [14].

### 10.1. Anti-Cancer Properties

Several corn phytochemicals have been proposed as potential preventive and chemotherapeutic agents against certain types of cancer. However, their chemical diversity and bioactivity are as diverse as the etiology and natural history of each type of cancer [145]. Corn carotenoids (e.g., violaxanthin, zeaxanthin, lutein, α-cryptoxanthin, and β-cryptoxanthin; yellow-to-orange varietals), ascorbic acid (white varietals), and phenolic compounds (e.g., anthocyanins, proanthocyanidins, phenolic acids; dark varietals) improve cellular redox status, correct DNA synthesis, intracellular signaling, and immunomodulation in normal cells while promoting cell growth arrest and apoptosis and reduce angiogenesis in cancer cells, in a QSAR-dependent manner [40,152].

Dark corn kernels (and other non-edible plant parts such as silk) have been the most studied since anthocyanins (and their phenolic precursors) from these and other plant sources possess fundamental anti-cancer molecular mechanisms (Table 3). For example, anthocyanins from alkali-treated (tortilla; rich in alkylated anthocyanins)> raw (rich in acylated anthocyanins) blue corn (Mixteco varietal) reduce cell viability (30–50% reduction), induce cell cycle arrest (at G1-S), and promote apoptosis in androgen-sensitive triple-negative breast (MDA-MB-453) and prostate (LNCaP) cancer cells, particularly when combined with 2-amino-N-quinolin-8-yl-benzenesulfonamide [99]. Al-Oqail et al. [100] demonstrated that corn silk phytochemicals (mainly) reduce cell viability and induce apoptosis (downregulation (bcl-2), upregulation (p53, Bax, caspase-3/9)) in breast cancer (MCF-7) cells in a dose-dependent manner via ROS-mediated mitochondrial pathway.

Also, purple corn anthocyanins (particularly Cy3G) inhibit 7,12-dimethylbenz[a]anthracene (DMBA)-induced mammary tumor development in both cancer-prone transgenic (human c-Ha-ras proto-oncogene, Hras128) and wild-type mice by pro-apoptotic mechanisms (caspase-3 upregulation and lower Ras protein levels) [101].

### 10.2. Anti-Diabetic Properties

Various anti-diabetic phytoremedies have been extensively used in folk medicine in many countries. The increasingly better understanding of the molecular mechanisms involved in the onset, progression, and fate of this NCCD has helped to identify “exclusive benefits” of novel corn phytochemicals in a structure-dependent manner. Inhibition of digestive enzymes, delay (gastrointestinal tract) or stimulation (peripheral tissues) of cellular glucose uptake/transport, insulinotropic and insulin-mimetic effects, and improved intracellular signaling are just some complementary mechanisms [152].

Notably, studies supporting the anti-diabetic/hypoglycemic action of corn phytochemicals from a QSAR standpoint are summarized in Table 2. Ranilla et al. [43] characterized 22 Peruvian corn landraces corresponding to five varietals (Arequipeno, Cabanita, Kulli, Granada, and Coruca) in terms of their antioxidant profile and inhibitory activity vs. α-glucosidase and α-amylase, which turns to be high (32.5–76.1% at 25 mg sample dose; 13.6–29.0 at 250 mg sample dose) in a varietal-specific (↑ dark varietals) and chemical structure (↑ anthocyanins rich varietals) dependent trend. Similarly, Smorowska et al. [40] evaluated the anti-diabetic potential of three blue (Chih 365, 367, and 503) and two yellow (Opoka, Kuskun) corn cultivars, confirming that the α-amylase inhibition capacity is higher in blue than yellow varietals, potentially due to the presence of anthocyanins (cyanidin (C3G) and peonidin (P3G)-3-O-glycosides) more than phenolic acids (caffeic, ferulic, and gallic acids).

Also, Luna-Vital and de Mejia [81] used dual-layer cell cultures (Caco-2 (intestinal) cells, INS-1E (pancreas) or HepG2 (hepatic) cells) to test the insulinotropic effect of C3G-rich hydroalcoholic extract obtained from purple corn pericarp, documenting a concurrent higher insulin secretion and hepatic glucose uptake via free fatty acid receptor-1 and glucokinase expression. Based on these and other in vitro studies, preclinical studies (animals), and controlled clinical trials (humans), a more significant number of patents exploiting the anti-diabetic effects of dark corn varieties are expected in the following years [152].

## 11. Habanero pepper (*Capsicum chinense* Jacq.)

The habanero pepper (Capsicum chinense Jacq.) is typically green when immature but changes color to yellow, orange, and red as it ripens (see Figure 1). In Mexico, it is primarily grown in the Yucatán region. About 80% of habanero pepper production is sold as fresh fruit, while the remaining 20% is used to produce sauces. Habanero peppers are significant in culinary applications due to their intense heat and distinctive flavor, which are essential in Mexican and Latin American cuisine. Most of these peppers are exported to the United States, Japan, South Korea, Italy, and Germany [153].

Capsaicin is the main phytochemical found in habanero peppers, with capsaicinoids and quercetin as minor components [154]. Moderate consumption of habanero peppers has been linked to anti-diabetic and anti-cancer effects. However, further clinical studies are recommended, particularly clinical trials focusing on habanero peppers’ anti-diabetic and anti-cancer potential and their phytochemicals.

### 11.1. Anti-Cancer Properties

The anti-cancer properties of habanero pepper have been explored and documented through cell line protocols and in vivo studies from ethanolic/methanolic extracts of fruits and leaves (Table 3). Evidence suggests that habanero pepper extract shows better anti-cancer activity in vitro than pure capsaicin. The reported mechanism includes anti-inflammatory effects, decreased ROS, the inhibition of electron transport from NADH to ubiquinone, or the direct link with Coenzyme Q [27,114].

### 11.2. Anti-Diabetic Properties

The anti-diabetic properties of habanero pepper are primarily attributed to capsaicin in prepared ethanolic extracts and sauces. In vitro and molecular docking studies have suggested that the mechanisms by which habanero pepper aids in blood sugar control include the inhibition of the glycolytic enzymes α-amylase and α-glucosidase [37,46]. Additionally, capsaicinoids have also been recognized for their anti-diabetic effects. A clinical study by Kenig et al. [67] concluded that moderate daily consumption of 4.4 mg of capsaicinoids can help lower glucose levels.

## 12. Prickly Pear (*Opuntia ficus-indica* L.)

Prickly pear (*Opuntia ficus-indica* L.), commonly known as Indian fig or nopal, is native to Mexico but is extensively cultivated in Central and South America, Australia, the Mediterranean basin, and South Africa. The fruits exhibit a range of colors, including green, white, yellow, orange, red, and purple, attributable to betalain-type pigments.

This plant flourishes in arid and semi-arid regions, with Mexico achieving a production volume of approximately 872.33 thousand metric tons in 2022, reflecting a 1% increase from the previous year [155]. Cladodes are fiber-rich and can be utilized as animal feed or for human consumption. At the same time, flavorful fruits are packed with valuable compounds such as polyphenols, dietary fibers, vitamins, and amino acids.

The growing demand for prickly pear is linked to its numerous health benefits, including anti-inflammatory, hypoglycemic, and antimicrobial [156]. Its antioxidant potential makes it a promising ingredient for innovative food products and natural extracts in food, pharmaceutical, and cosmetic applications.

### Anti-Cancer and Anti-Diabetic Properties

Recent research has documented and summarized the bioactive properties of *Opuntia ficus-indica* [156]. However, studies confirming its anti-diabetic and anti-cancer effects remain limited (Table 2 and Table 3). The anti-cancer potential of *Opuntia ficus-indica* has primarily been observed using chloroform, acetone, and methanol extracts, as well as juices derived from prickly pear, which have been assessed in various cancer cell lines. One notable study investigated the anti-cancer properties of nine different juices extracted from Mexican prickly pear varieties against colon, prostate, breast, and liver cancer cell lines. The Moradillo variety showed the most promising in vitro effects on prostate cancer cells, significantly reducing cell viability [111].

Further findings suggest that extracts from Opuntia may lower cell viability by stimulating apoptosis pathways, inhibiting cyclooxygenase-2, and promoting a Bax/Bcl2 ratio ([112]; see Table 2). Conversely, the anti-diabetic effects of *Opuntia ficus-indica* have been demonstrated in both the fruit (prickly pear) and the cladodes (nopal) in models involving streptozotocin-induced diabetic rats. The mechanisms behind its anti-diabetic properties include a reduction in intestinal glucose absorption and enhanced peripheral glucose uptake via the activation of the AMPK pathway [85,86], see Table 1).

## 13. Agave (*Agave* spp. L)

Agave, also known as Maguey (*Agave* spp. L., Asparagaceae; derived from the Greek word agauós, meaning “nobleman”), is a perennial monocotyledonous plant characterized by a short stem and thick, fleshy, sword-sized leaves (commonly referred to as pencas) arranged in rosettes. These leaves are attached to an underground ovoid stem known as the pine or head [157].

In Mexico, Agave production has seen a 14% increase from 2016 to 2024, achieving an export value of approximately USD 1380.21 million [158]. This resilient plant flourishes in rocky, arid environments at altitudes between 1700 and 2400 m above sea level, demonstrating an impressive tolerance to extreme conditions such as prolonged drought. Notably, 87% of all known Agave species (n = 310) are indigenous to Mexico [159].

Numerous Agave species, including Americana, Angustifolia, aplanatic, astrovirus, create, duranguense, fourcroydes, inaequidens, Karpinski, Lechuguilla, mapisaga, Maximilian, rhodacantha, palmeri, salmiana, tequila, and Valenciana, are utilized in the production of both distilled and non-distilled beverages. These include tequila and raicilla (from Jalisco/Nayarit), mezcal (from Oaxaca), sotol (from Chihuahua), bacanora (from Sonora), and pulque (from central and southern Mexico). These species have recently been explored for their potential to yield fructan-rich syrups [159].

Moreover, the industrial processing of Agave results in various underutilized by-products, such as leaves, bagasse, and fibers. These by-products are rich in bioactive compounds with health-promoting and technological properties, rendering them valuable to the food, cosmetic, and pharmaceutical industries. The agave pine and bagasse are rich in policosanols, sapogenins/saponins (hecogenin, thiogenin, and canthalasaponin 1, Magueyoside B, kammogenin glycosides), and diverse flavonoids; its leaves are in phenolic compounds (phenolic acids (hydroxybenzoic/hydroxycinnamic), flavonoids (kaempferol and quercetin, hecogenin, diosgenin, chlorogenin, kammogenin, and gentrogenin), hydrolyzable and condensed (proanthocyanidins) tannins and lignans) and steroidal saponins (Kammogenin glycosides and aglycones, manogenin glycosides) [102,157].

### 13.1. Anti-Cancer Properties

The evidence of agave bioactives against cancer remains scarce. However, saponins/sapogenins appear more promising than other agave phytochemicals (Table 3). Santos-Zea et al. [102] demonstrated that the presence of specific saponins (Magueyoside B, gentrogenin tetraglycosides) > sapogenins present in concentrated sap (aguamiel) from various Agave species is responsible for the antiproliferative activity of this product in colon cancer cells. Álvarez-García et al. [103] provided compiling evidence on the synergistic anti-cancer potential of *A. salmiana fructans* + chicory inulin mixtures on reducing the viability/proliferation (by pro-apoptotic mechanisms and short-chain fatty acid production) of colon cancer (HT29/SW480) cells with no apparent effects on healthy (CRL1831) cell counterparts.

### 13.2. Anti-Diabetic Properties

Research on the anti-diabetic potential of bioactive compounds from Agave species is limited, but promising results are emerging. The combined effects of phenolic compounds and fructans appear beneficial, while saponins and sapogenins contribute to lipid-lowering effects, which are important in the later stages of type 2 diabetes mellitus (T2DM).

Aleem et al. [52] found that a methanolic extract rich in phenolic compounds from the leaves of *Agave americana var. marginata* normalized blood glucose and exhibited antioxidant and anti-inflammatory effects in alloxan-induced diabetic rats, also reducing depression-like behaviors.

Leal-Díaz et al. [82] examined the effects of aguamiel from *Agave salmiana* and its extracted saponins in obese mice. They reported that aguamiel reduced obesity-related issues, dyslipidemia, and glycemic disturbances while improving fecal microbiota. Saponins also reduced insulin resistance and enhanced fatty acid oxidation gene expression.

## 14. Bee Honey (*Apis mellifera*)

Honey is a thick nectar primarily composed of sugars, water, and various other components, including enzymes, amino acids, organic acids, carotenoids, vitamins (notably B6, thiamine, niacin, riboflavin, and pantothenic acid), minerals, and aromatic substances. It is collected through apiculture, which involves breeding and harvesting honeybees (*Apis mellifera* L.), or meliponiculture, the practice of breeding and utilizing native stingless bees (*Melipona bees*), from a range of plants found in tropical and subtropical ecosystems [160].

The global honey industry is primarily driven by the rising consumer demand for natural, premium organic, and locally sourced food products. In 2022, the global honey market was valued at USD 9.32 billion [161]. Mexico is the ninth largest honey producer worldwide, reporting 63,362 tons in 2021. The country’s rich biodiversity provides honey with various unique biological properties. The significance of honey in Mexico dates to pre-Hispanic times when the Mayans cultivated the Trigona and Melipona varieties. At that time, honey was used as food, a medicinal remedy, and in various rituals. Today, it serves as a natural sweetener for foods and beverages while retaining its traditional folk medicinal uses, being considered beneficial for throat issues and wound healing [162].

### 14.1. Anti-Cancer Properties

The anti-cancer potential of honey has been demonstrated through in vitro studies on cancer cell lines (see Table 3). Key phytochemicals in honey include sphingoid, phytosphingosine, sphinganine, and various polyphenols. Research indicates that honey can trigger the apoptosis pathway, leading to an increased expression of pro-apoptotic proteins such as Apaf1, caspase-9, IFN-c, IFNGR1, and p53. Simultaneously, it reduces the expression of anti-apoptotic proteins, including E2, ESR1, TNF-α, COX-2, and Bcl-xl1, while also modulating neo-angiogenesis [71,117]. Overall, studies suggest that honey provides substantial evidence of various anti-cancer mechanisms, indicating its strong potential for oncological applications.

### 14.2. Anti-Diabetic Properties

The anti-diabetic properties of bee honey have been demonstrated in both in vitro and in vivo studies (on rodents) (see Table 2). These properties are linked to the presence of phenols, flavonoids, and polysaccharides (refer to Table 1). Fundamental mechanisms of action include the inhibition of α-amylase and α-glucosidase, an increase in the activity of SOD, GSH, and CAT enzymes, as well as a reduction in blood lipids and oxidative stress [28,50,61].

## 15. Xoconostle (*Opuntia joconostle*)

Xoconostle (*Opuntia joconostle*) is a genus within the Cactaceae family that comprises approximately 300 species. This genus is native to North America, with its species thriving in arid and semi-arid climates. In Mexico, around 78 species are endemic [163]. Opuntia plants produce both sweet fruits (cactus pears) and sour fruits (xoconostles) (Figure 1), which are considered valuable food sources in Latin America (Morales et al., 2012). The most cultivated and marketed species is *O. joconostle* F.A.C, followed by *O. matudae Scheinvar cv*. Rosa.

Xoconostles consist of three parts: the epicarp (skin), the mesocarp (flesh), and the endocarp (where the seeds are densely packed in a mucilaginous structure) [30]. The edible portion is valued for its vibrant color, ranging from pink to dark purple, and its acidic flavor. They can be enjoyed raw or used as a condiment in sauces and other Mexican dishes and in the production of candies, jellies, and beverages.

In folk medicine, xoconostles have been used to treat conditions such as T2DM, hypertension, obesity, and respiratory issues, mainly due to their bioactive compounds [29]. Phytochemical studies have revealed that xoconostles contain various pigments, including betalains (betanidin derivatives), flavonoids (such as isorhamnetin, quercetin, and kaempferol glycoside derivatives), and phenolic acids (like eucomic acid and ferulic acid hexoside). They are also rich in soluble fiber, minerals, polyunsaturated fatty acids, and tocopherols [164]. Few studies have been reported based on probing the anti-cancer and anti-diabetic potential. Thus, based on their phytochemistry, it is recommended that studies be carried out based on them.

### 15.1. Anti-Cancer Properties

In vitro studies have shown that the aqueous extract of Xoconostles epicarp has an antiproliferative effect on two breast cancer cell lines, MCF-7 and MDA-MB-231. This effect leads to the arrest of cells in the G2/M phase of the cell cycle [119] (Table 3). However, there is limited evidence regarding the anti-cancer potential of Xoconostles. Future studies should be conducted in vitro using cell lines and in vivo using rodent models to strengthen the scientific evidence.

### 15.2. Anti-Diabetic Properties

Table 2 summarizes the anti-diabetic potential of Xoconostles. A clinical trial showed that the peel of Opuntia Xoconostle significantly reduced serum glucose levels and increased insulin levels in patients with T2DM [87]. Experimental studies on streptozotocin-induced T2DM rats confirmed a decrease in glucose concentration after administering aqueous extracts from the mesocarp of *O. joconostle* fruit [88]. More recently, the anti-diabetic activity of *O. matudae* fruit was demonstrated in studies involving streptozotocin-induced diabetic mice and a hepatic glucose output model [29].

## 16. Pomegranate fruit (*Punica granatum*)

Pomegranate (*Punica granatum*) (Figure 1) is a member of the Lythraceae family and is native to the Middle East and Mediterranean regions. Today, it is cultivated in numerous countries for its nutritional and medicinal benefits, including tropical and subtropical areas in Mexico. The global pomegranate market was valued at USD 221.32 million in 2023 and is projected to reach USD 375.68 million by 2031, growing at a compound annual growth rate (CAGR) of 9.23% from 2024 to 2031 [165].

Various parts of the pomegranate plant, including seeds, roots, bark, wood sprouts, leaves, flowers, and fruit, have been utilized [166]. The primary uses of pomegranate—particularly its seeds, peels, and juice—include producing a range of processed foods such as beverages, jellies, and jams. Recently, pomegranate has gained recognition as a superfood, and research has explored its role as a functional food and nutraceutical source. 

The bioactive compounds identified in pomegranate include alkaloids, anthocyanins, tannins, flavonoids, phenolics, proanthocyanidins, sterols, terpenes, xanthonoids, fatty acids, organic acids, lignans, saccharides, and vitamin C [166].

### 16.1. Anti-Cancer Properties

The anti-cancer potential of pomegranate fruit and its derivatives has been extensively documented in vitro, with studies focusing on various types of cancer, including prostate, colorectal, leukemia, pancreatic, hepatocellular, skin, and lung cancer. These effects have also been observed in vivo in animal models and human clinical trials (Table 3; [167]).

The anti-cancer effects are linked to a high content of polyphenols, such as punic acid, ellagic acid, and anthocyanins (including delphinidin, cyanidin, and pelargonidin), as well as ellagitannins like punicalagin and flavonoids such as luteolin, kaempferol, and quercetin [166].

The mechanisms behind pomegranate fruit’s anti-cancer activity include the induction of apoptosis, the arrest of the cell cycle, and the modulation of key signaling pathways, including nuclear factor-erythroid factor 2-related factor 2 (Nrf2), nuclear factor kappa B (NF-kB), and mitogen-activated protein kinase (MAPK) [166].

### 16.2. Anti-Diabetic Properties

The anti-diabetic potential of pomegranate fruit, peel, and seed oil has been documented in various studies. Most literature focuses on in vivo studies using rodent models and clinical trials (Table 2). For example, a study by Parmar and Kar [59] reported that treating alloxan-induced diabetic rats with 200 mg of pomegranate methanol peel extract for 10 days decreased fasting serum glucose and increased insulin levels, along with anti-lipid peroxidation effects. Another study by Das et al. [53] found that administering 300 mg/kg of methanolic seed extract of pomegranate significantly reduced blood glucose levels by 47% in STZ rats. Chakraborty et al. [57] also demonstrated that giving 3 mL of pomegranate fruit juice per rat for four weeks reduced serum glucose levels in alloxan-induced diabetic rats by increasing insulin levels.

The anti-diabetic properties of pomegranate are attributed to various phytoconstituents, including phenolic acids such as gallic acid, caffeic acid, chlorogenic acid, ferulic acid, and coumaric acid; tannins, particularly ellagitannins (punicalagins and granatins) and gallotannins; as well as flavonoids like catechin, quercetin, and phloridzin. The mechanisms through which pomegranate exerts its anti-diabetic effects are linked to enhanced antioxidant activity and improved insulin secretion.

## 17. Onion and Garlic (*Allium cepa* L. and *Allium sativum*)

The Allium genus consists of a group of flowering plants belonging to the Amaryllidaceae family, which includes over 850 species cultivated as vegetables and ornamental plants. Due to their substantial annual production and consumption, the most significant vegetables within the Allium species are onions (*Allium cepa*) and garlic (*Allium sativum*). In 2021, the global market size for Allium species was valued at USD 51,920 million, and it is projected to reach USD 64,092.58 million by 2032, with a compound annual growth rate (CAGR) of 2.1% during the forecast period [168].

In Mexico, Allium species are commonly used in soups and are key ingredients in various traditional dishes. Additionally, these plants have been utilized in traditional medicine since ancient times due to their bioactive components, which include alliin, allicin, ajoene, sterols, organosulfur compounds, flavonoids, polyphenols, and polycarboxylic acids [124,169].

### 17.1. Anti-Cancer Properties

Extracts rich in phytochemicals from Allium species have demonstrated the ability to influence cancer-associated pathways. Specifically, *Allium cepa* (onions) has been examined for its phytochemical-rich extracts, primarily obtained through ethanolic and hydroalcoholic extractions. Onion extracts are notable sources of quercetin and alliin derivatives, which show anti-cancer potential against various types of cancer (see Table 3).

In addition, multiple anatomical parts of onions (bulbs and cloves) and garlic (including aged, purple, and black varieties) have been studied for their phytochemical content and anti-cancer potential. Diallyl disulfide is the primary organosulfur compound found in garlic [170]. Other compounds, such as alliin, allicin [125], S-allyl-L-cysteine [127], thiosulfate [129], and phenolic acids [125], have also been identified as bioactive constituents with anti-cancer properties in garlic.

Research has primarily focused on the anti-cancer activity of garlic extracts against breast, colon, and lung cancers, utilizing in vitro cell line models [128,129,130]. The anti-cancer mechanisms identified include the upregulation of pro-apoptotic proteins and genes, activation of antioxidant enzymes, and depolarization of mitochondrial membranes. Moreover, garlic extracts have been shown to enhance the effectiveness of cancer treatments, such as 5-fluorouracil, doxorubicin, and oxaliplatin [129] (see Table 2).

### 17.2. Anti-Diabetic Properties

The anti-diabetic potential of Allium species has been supported by clinical trials and in vitro studies (see Table 2). A randomized clinical trial involving 65 patients with T2DM found that supplementation with aged garlic capsules (2400 mg) decreased the cardio-ankle vascular index and improved endothelial function [65].

Additionally, a recent study highlighted the enzymatic inhibitory effects of aqueous extracts from garlic and onions (both purple and white), which are rich in phenolic acids and allicin compounds, including diallyl-, methyl allyl-, and allyl methyl-thiosulfate ([44]; see Table 2). These aqueous extracts inhibited the activity of α-amylase and α-glucosidase in a dose-dependent manner, with concentrations ranging from 0 to 4 mg/mL. Notably, the phenolic-rich extract from white onion had a more significant inhibitory effect on α-amylase. In contrast, garlic extract demonstrated a more robust capability for inhibiting α-glucosidase activity in vitro.

## 18. Pumpkin Flower (*Curcubita pepo*)

The pumpkin flower (*Cucurbita pepo*) is part of the Cucurbitaceae family, which encompasses around 118 genera and 825 species, primarily found in tropical regions. This family is most recognized for its fruits, which exhibit various sizes, shapes, and colors. Notable examples include pumpkins, gourds, squashes, watermelons, melons, courgettes, and cucumbers. The fruits, seeds, and flowers of *C. pepo* are edible but also have applications in traditional medicine and various industries.

The processed pumpkin market, which includes dried products, purées, and concentrates, was valued at USD 1.34 billion in 2020. It is expected to grow at a compound annual growth rate (CAGR) of 6.5% from 2021 to 2028 [171].

*Cucurbita pepo* L., commonly known as squash, is native to Mexico and has long been a staple in the human diet ([172]. The immature fruit is enjoyed as a vegetable, while the mature fruit offers a sweet flavor and is often used in desserts. Roasted seeds can be incorporated into baked goods or mixed with honey to create a treat called “palanquetas”. Additionally, flower buds and blossoms are used to prepare “quesadillas”.

### Anti-Cancer and Anti-Diabetic Properties

*C. pepo* has long been utilized in traditional medicine across various countries to treat various ailments. However, no scientific evidence supports its anti-cancer properties, and research on its anti-diabetic potential is limited. An ethanol extract of C. pepo flowers demonstrated significant inhibition of α-glucosidase, with an IC50 value of 144.77 µg/mL, and exhibited a dose-dependent hypoglycemic effect in healthy mice [39]. The extract’s phytochemical analysis revealed key compounds, including (+)-catechin, (-)-epicatechin, rutin, syringic acid, hesperidin, and quercetin 3-O-glucoside [39].

## 19. Flor de Cempasúchil (*Tagetes erecta*)

The Tagetes genus, belonging to the Asteraceae family, comprises 56 species distributed across North and South America, with 24 to 30 species found in Mexico. One prominent species is *Tagetes erecta* L., commonly referred to as the Aztec marigold or “Flor de cempasúchil” in Spanish. This flower holds significant cultural importance during Mexico’s “Day of the Dead” celebration.

The flowers of *T. erecta* are utilized in gastronomy as an ingredient in salads and a natural food colorant. Several studies have shown that *T. erecta* exhibits a variety of biological activities, including anti-diabetic and anti-cancer properties [35,72,122].

### 19.1. Anti-Cancer Properties

Table 3 illustrates the anti-cancer potential of *T. erecta*. The hydroalcoholic extract from its flowers demonstrates notable anti-cancer activity against ovarian carcinoma cells, primarily attributable to the predominant polyphenol, lacritirin [122]. Additionally, this extract exhibits cytotoxic effects on Lewis lung carcinoma (LLC1) and human breast carcinoma (MCF-7) cell lines, leading to reduced tumor growth and enhanced effectiveness of etoposide in a xenograft lung cancer model. Essential compounds found in the extract include 2,3-dihydrobenzofuran, octadecanoic acid, and oleic acid [72].

Moreover, essential oils from *T. erecta* improve redox status and immune function, potentially lowering cancer risk in rats with MNNG-induced gastric cancer. This mechanism involves inhibiting pro-inflammatory cytokines and modulating the expression of several mRNA factors, including caspase-3, Bax, and Bcl-2. Phytochemical analysis revealed that limonene, cis-ocimene, and (+)-2-carene are significant compounds [70].

### 19.2. Anti-Diabetic Properties

Table 2 presents the anti-diabetic potential of *T. erecta*. A recent study revealed that the ethanolic extract of the plant inhibits α-glucosidase, with an IC50 value of 201.83 ± 38.89 μg/mL, which is more effective than acarbose (IC50 = 297.21 ± 15.81 μg/mL). Additionally, this extract significantly prevents the formation of advanced glycation end-products, demonstrating an IC50 of 47.19 ± 17.71 μg/mL. The phytochemical analysis identified several critical bioactive compounds, including hyperoside, isoquercitrin, quercetin, ellagic acid, and vanillic acid, underscoring their potential in the management of T2DM [35]. Furthermore, another in vitro study highlighted the anti-diabetic properties of quercetagetin, derived from a 70% ethanol–water extract of inflorescences, which also inhibits α-glucosidase [35].

## 20. Conclusions: Limitations and Future Research

Mexico is a geographic region known for its rich agrobiodiversity. The country produces a variety of plants and plant-based foods that are abundant in phytoconstituents with notable health benefits. Critical sources of these phytochemicals include fruits, seeds, grains, cereals, and other plants, which often exhibit diverse properties such as anti-cancer and anti-diabetic effects. These benefits have been demonstrated in vitro using various cancer cell lines and in vivo in animal models with specific diseases.

However, there are limitations in applying these findings to industrial, nutraceutical, or pharmaceutical uses. The phytochemical content and bioactive properties can vary significantly between and within species, influenced by cultivation in different regions. Factors like extraction methods, quantification protocols, and environmental conditions (soil, climate, and nutrients) all contribute to these variations.

Moreover, scientific information on specific plants is lacking. Species such as *Tagetes erecta*, *Cucurbita pepo*, *Opuntia joconostle*, *Opuntia ficus-indica*, and *Capsicum chinense* are notable for their phytochemical content. However, their anti-diabetic and anti-cancer properties (and the mechanisms behind them) remain underexplored.

Additionally, current research on the consumption of these plant-based foods and their isolated constituents regarding their anti-diabetic and anti-cancer effects in human clinical trials is limited. Therefore, developing clinical trials in humans is crucial for future research.

## Figures and Tables

**Table 1:** Protocols and phytochemical screening for key plants and plant-based foods derived from Mexican agrobiodiversity.

Mexican Plant and Plant-Based Foods	Extraction Method	Protocol for Quantification/Identification	Phytochemical Content	Reference
Cocoa (*Theobroma cacao* L.)	Ultrasonication	Spectrophotometric method	-Total polyphenols: 0.67–5.67 (g GAE/100 g dw) -Theobromine: 6.86–17.46 (mg/g) -Caffeine: 0.25–1.03 (mg/g)	[20]
Coffee (*Coffea arabica* L. and *C. canephora* R.)	Maceration	High-Performance Liquid Chromatography (HPLC)	-Chlorogenic acid: 14.5–55 (mg/g) -Caffeine: 1.8–29 (mg/g)	[21]
Avocado (*Persea americana Mill*)	Soxhlet	Gas Chromatography/Mass Spectrometry (GC-MS)	-Avocatins: 32.28 (µg/g) -Long-chain fatty acids: 39.66 (µg/g) -Polyhydroxy fatty acids: 24.26 (µg/g)	[22]
Vanilla (*Vanilla planifolia*)	Maceration	High-Performance Liquid Chromatography (HPLC)	-Vanillin: 7.6–14.26 (g/dw)	[23]
Mango (*Mangifera indica* L.)	Maceration	Spectrophotometric method	-Total polyphenols: 37 (mg GAE/g) -Total flavonoids: 19.3 (mg QE/g) -Mangiferin: 5523 (µg/g)	[24]
	Ultrasonication	Spectrophotometric method/HPLC	-Total polyphenols: 9.87 (mg GAE/g) -Flavonoids: 0.37 (mg GAE/g) -Ascorbic acid: 959 (mg GAE/g) -Shikimic acid: 106.9 (µg/g) -Chlorogenic acid: 1.54 (µg/g) -Epicatechin: 9.29 (µg/g) -Vanillin: 15.11 (µg/g)	[19]
	Ultrasonication	UPLC-DAD	-Mangiferin: 1259 -Catechin: 75 -Syringic acid: 12	[18]
	Ultrasound-microwave-assisted extraction	Spectrophotometric method	-Total polyphenols: 54.15 (mg GAE/g dw)	[16]
Corn (*Zea mays* L.)	Ultrasonication	Spectrophotometric method	-Anthocyanins: 36.05 (mg of TA/100 g)	[14]
	Maceration	Spectrophotometric method/HPLC	-Total polyphenols 349,39–485,71 (mg GAE/100 g) -Total flavonoids: 24.00–105 (mg CE/100 G) -Anthocyanins: 1.38–74.90 (mg/100 g) -Ferulic acid: 103.34–164.90 (mg/100 g) -Coumaric acid: 5.57–15.52 (mg/100 g)	[25]
Habanero pepper (*Capsicum chinense* Jacq.)	Maceration	UPLC-DAD	-Total polyphenols: 2414.92 (mg 100 g/dw) -Chlorogenic acid: 311.13 (mg 100 g/dw) -Myricetin 489.33 ± 16.57 (mg 100 g/dw) -Quercetin + Luteolin 505.30 ± 0.79 mg (mg 100 g/dw)	[26]
	Maceration	HPLC	-Capsaicinoids 7.64 (mg 100 g/dw)	[27]
*Apis mellifera*	Ultrasonication	Spectrophotometric method	-Total polyphenols: 29.91 (mg QE/100 g) -Total flavonoids: 1.92 (mg QE/100 g)	[28]
	High hydrostatic pressure	Spectrophotometric method	-Total polyphenols 29.89 (mg GAE/100 g) -Carotenoids: 0.406 (mg/100 g)	[13]
Prickly pear (*Opuntia ficus-indica* L.)	Ultrasonication	High-Performance Liquid Chromatography (HPLC)	-Total betalains: 310–2580 (µg/g dw)	[17]
Agave (*Agave lechugilla*)	Ultrasonication	High-Performance Liquid Chromatography (HPLC)	-Isorhamnetin: 750.74–1251.96 (μg/g dw) -Flavanone: 291.51–214.10 (μg/g dw) -Hesperidin: 34.23 (μg/g dw) -Delphinidin: 24.23 (μg/g dw) -Quercetin: 15.57 (μg/g dw) -Kaempferol: 13.71 (μg/g dw) -Cyanidin: 12.32 (μg/g dw) -Apigenin: 9.70 (μg/g dw) -Catechin: 7.91 (μg/g dw)	[15]
Xoconostle (*Opuntia joconostle* W.)	Maceration	Spectrophotometric method/HPLC	-Isorhamnetin-3-O-rutinoside: 0.526 (mg/g)	[29]
	Maceration	Spectrophotometric method	-Total polyphenols: 38.57–50.43 (mg GAE/g) -Total flavonoids: 3.93–24.18 (mg CE/g)	[30]
Xoconostle (*Opuntia matudae* S.)	Maceration	Spectrophotometric method	-Total polyphenols: 33–59 (mg GAE/g) -Total flavonoids: 0.86–58.40 (mg CE/g)	[30]
Pomegranate fruit (*Punica granatum*)	Maceration	Spectrophotometric method	-Total polyphenols: 1820–2450 (mg GAE/100 g dw) -Total flavonoids: 170–320 (mg QE/100 g dw)	[31]
Onion (*Allium cepa* L.)	Maceration	Spectrophotometric method	-Total polyphenols: 11.86–25.40 (mg GAE g/dw)	[32]
Garlic (*Allium sativum*)	Maceration	Spectrophotometric method	-Total polyphenols: 140.65 (mg GAE g/dw) -Total flavonoids 68.8 (mg GAE g/dw)	[33]
Pumpkin flower (*Curcubita pepo*)	Maceration	Spectrophotometric method	-Total polyphenols: 0.187–0.7 (mg GAE/g dw) -Total flavonoids: 0.025–0.37 (mg QE/g dw)	[34]
Flor de cempasúchil (*Tagetes erecta*).	Maceration	Spectrophotometric method	-Total polyphenols: 0.63–1.3 (mg GAE/g dw) -Total flavonoids: 3.5–22 (mg QE/g dw)	[34]
